# Supplementary material for: Association between Circulating Fibroblast Growth Factor 21 and Aggressiveness in Thyroid Cancer
Source: Cancers (Basel). 2019 Aug 12;11(8):1154. doi: 10.3390/cancers11081154 (PMC6721537; doi:10.3390/cancers11081154)
Supplement: Supplementary file 1 [file cancers-11-01154-s001.pdf]

Supplementary Materials

Association between Circulating Fibroblast Growth Factor 21 and Aggressiveness in Thyroid Cancer

Yea Eun Kang, Jung Tae Kim, Mi Ae Lim, Chan Oh, Lihua Liu, Seung-Nam Jung, Ho-Ryun Won, Kyungmin Lee, Jae Won Chang, Hyon-Seung Yi, Hyun Jin Kim, Bon Jeong Ku, Minho Shong and Bon Seok Koo

Table S1. Clinicopathologic parameters in patients with papillary thyroid carcinoma (PTC) (N = 127).

**Variables**		**Mean ± SD or Number of Patients (%)**
Sex	Male	18 (14.2)
	Female	109 (85.8)
Tumor size	≤2 cm	108 (85)
	>2 cm	19 (15)
Multicentricity	No	93 (73.2)
	Yes	34 (26.8)
Microscopic capsular invasion	No	41 (32.3)
	Yes	86 (67.7)
Extrathyroid extension	No	58 (45.7)
	Yes	69 (54.3)
Lymphovascular invasion	No	27 (21.3)
	Yes	100 (78.7)
Lymph node metastasis	No	85 (66.9)
	Yes	42 (33.1)
Central lymph node metastasis	No	85 (66.9)
	Yes	42 (33.1)
Lateral lymph node metastasis	No	114 (89.8)
	Yes	13 (10.2)
Recurrence	No	118 (92.9)
	Yes	9 (7.1)
Survival	No	4 (3.1)
	Yes	123 (96.9)
$BRAF^{V600E}$ mutation	No	3 (2.4)
	Yes	21 (16.5)
	Unknown	103 (81.1)
Follow-up period (months)		84.7 ± 37.4

Data are presented as means ± SDs or number of patients (%).

Table S2. Correlation analysis between serum FGF21 level and metabolic parameters in total participants with PTC (N = 127).

Variables	Mean ± SD or Number of Patients (%)	
Age	0.131	0.092
BMI (kg/m^2)	0.182	0.040*
Fasting glucose (mg/dL)	0.015	0.850
Triglycerides (mg/dL)	0.223	0.006**
Total cholesterol (mg/dL)	0.125	0.107
LDL cholesterol (mg/dL)	0.018	0.840
HDL cholesterol (mg/dL)	-0.140	0.017*
AST (IU/L)	0.053	0.498
ALT (IU/L)	0.042	0.590

Data are presented as means ± SDs or number of patients (%). Abbreviation: BMI, body mass index. LDL, low density lipoprotein. HDL, high density lipoprotein. AST, aspartate transaminase, ALT, alanine transaminase. a Coefficients (r) were calculated using Spearman's method. * *p*-value < 0.05, ** *p*-value < 0.01.

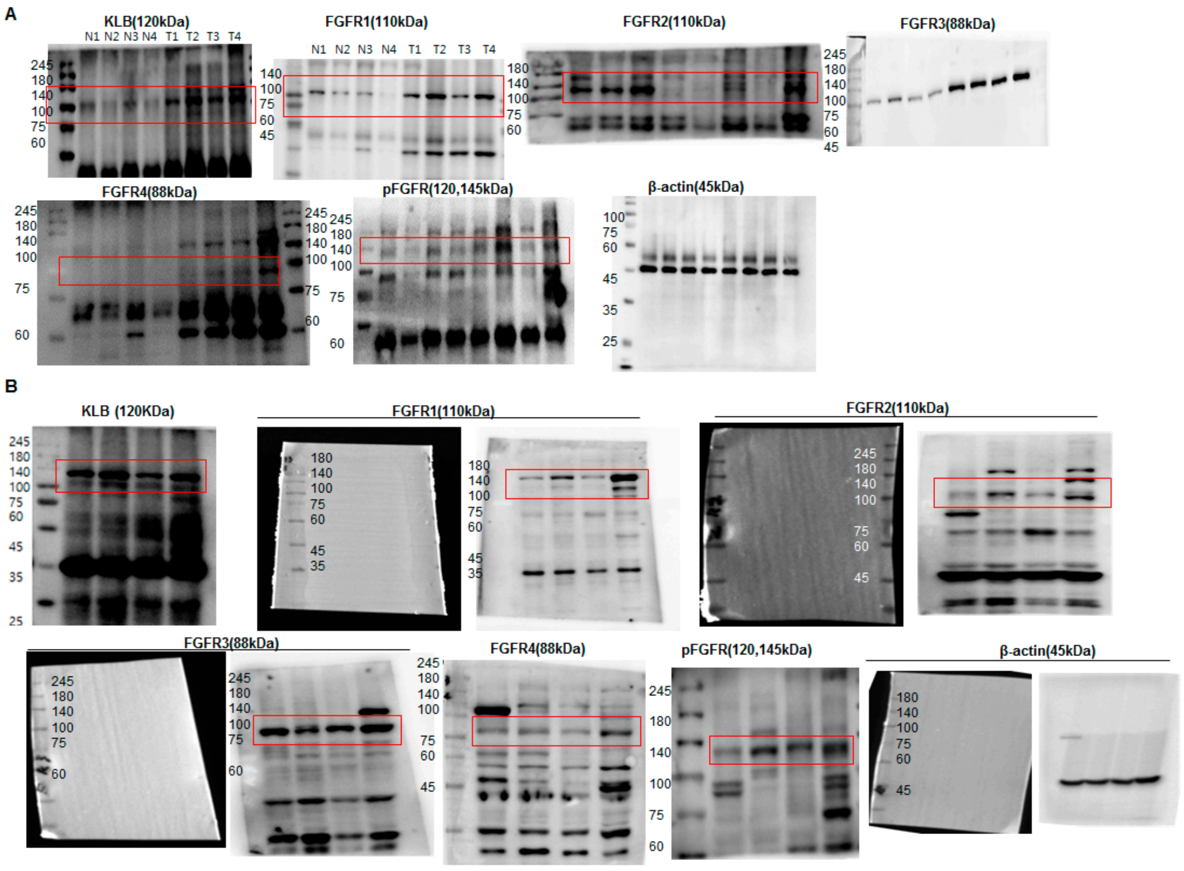

Figure S1. Raw data of Western blot analysis of Figure 3. (**A**) Raw data of Figure 3A. (**B**) Raw data of Figure 3B.

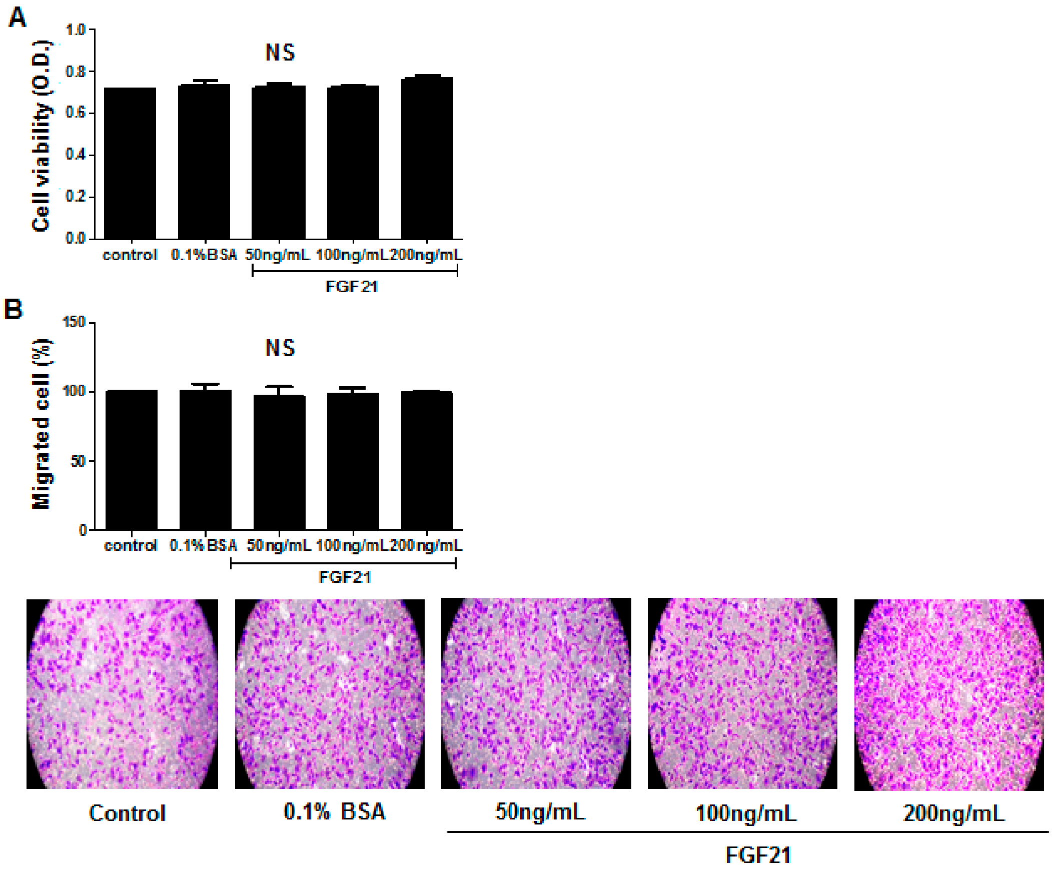

Figure S2. Effects of FGF21 on cell viability and migration of Nthy-ori3-1 cells. (**A**) Effects of FGF21 on cell viability in Nthy-ori3-1 cells treated with recombinant FGF21 (rFGF21) or 0.1% BSA negative control) for 12 h (**B**) Effects of FGF21 on migration in Nthy-ori3-cells treated with rFGF21.

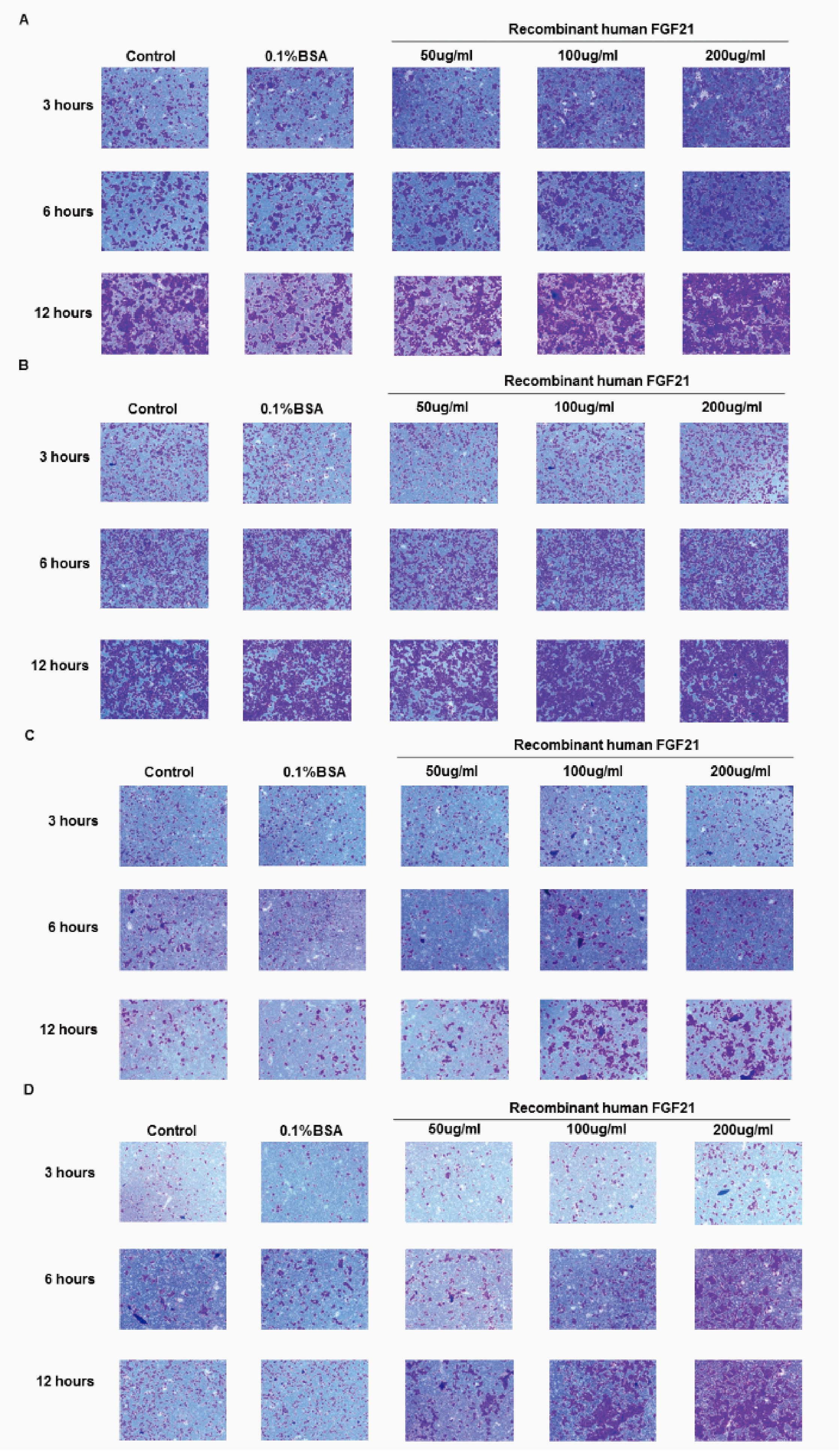
A
Recombinant human FGF21
Control
0.1%BSA
50ug/ml
100ug/ml
200ug/ml
3 hours
6 hours
12 hours
B
Recombinant human FGF21
Control
0.1%BSA
50ug/ml
100ug/ml
200ug/ml
3 hours
6 hours
12 hours
C
Recombinant human FGF21
Control
0.1%BSA
50ug/ml
100ug/ml
200ug/ml
3 hours
6 hours
12 hours
D
Recombinant human FGF21
Control
0.1%BSA
50ug/ml
100ug/ml
200ug/ml
3 hours
6 hours
12 hours

Figure S3. Effects of FGF21 on cell migration and invasion of PTC cells. (**A**) Effects of FGF21 on cell migration of BCPAP cells for 3, 6, and 12 hours. (**B**) Effects of FGF21 on cell migration of TPC-1 cells for 3, 6, and 12 hours. (**C**) Effects of FGF21 on cell invasion of BCPAP cells for 3, 6, and 12 hours. (**D**) Effects of FGF21 on cell invasion of TPC-1 cells for 3, 6, and 12 hours.

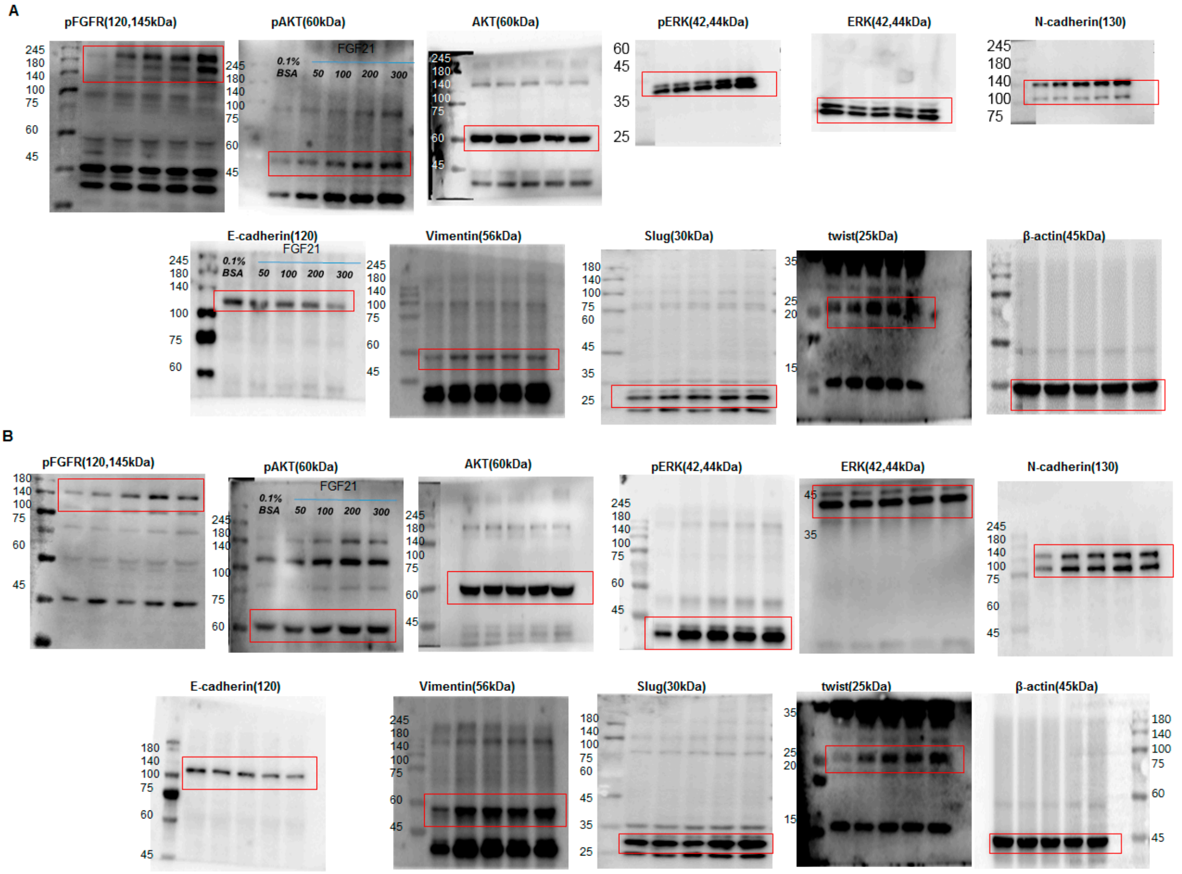

Figure S4. Raw data of Western blot analysis of Figure 4. (**A**) Raw data of Figure 4A. (**B**) Raw data of Figure 4B.

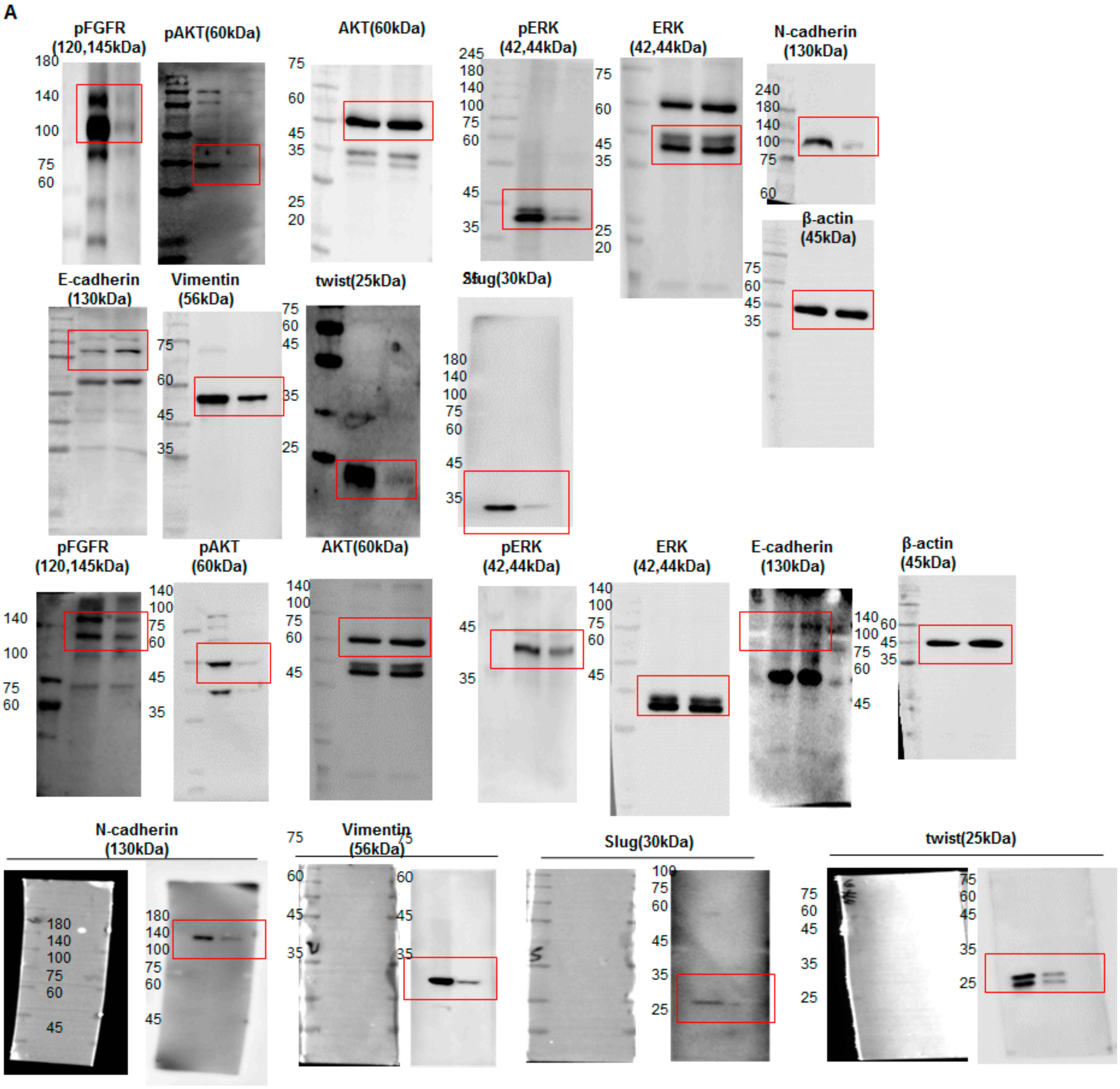

Figure S5. Raw data of Western blot analysis of Figure 5. (**A**) Raw data of Figure 5E. (**B**) Raw data of Figure 5F.

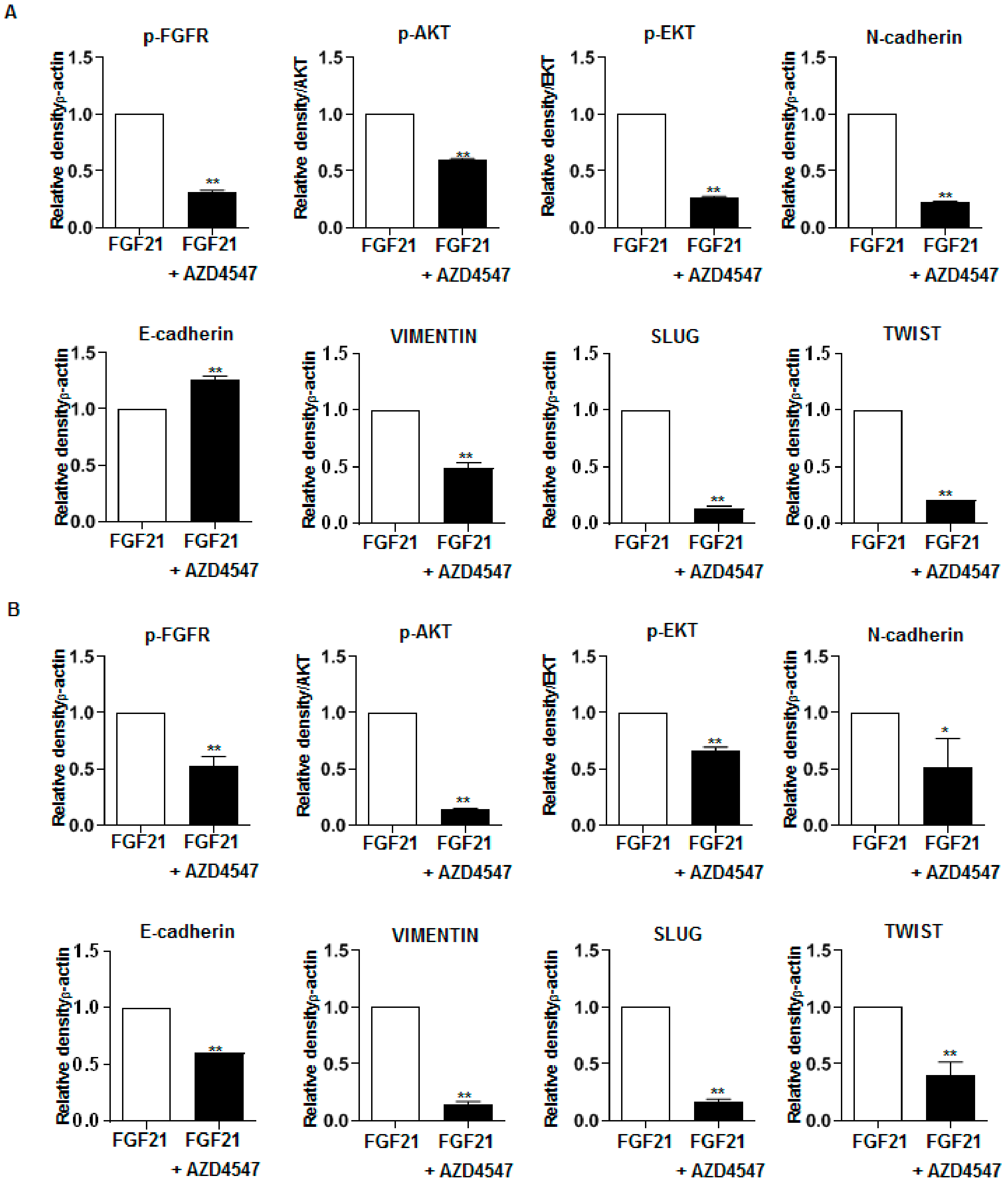

Figure S6. Comparison of relative density of Western blot analysis of Figure 5. (**A**) Comparison of relative density of Western blot analysis of Figure 5E. (**B**) Comparison of relative density of Western blot analysis of Figure 5F.

© 2019 by the authors. Licensee MDPI, Basel, Switzerland. This article is an open access article distributed under the terms and conditions of the Creative Commons Attribution (CC BY) license (http://creativecommons.org/licenses/by/4.0/).
